# Novel Gain-Optimized Two-Step Fusion Filtering Method for Ranging-Based Localization Using Predicted Residuals

**DOI:** 10.3390/s25092883

**Published:** 2025-05-02

**Authors:** Bo Chang, Xinrong Zhang, Na Sun, Hao Ni

**Affiliations:** 1 Faculty of Electronic Information Engineering, HuaiYin Institute of Technology, Huaian 223003, China; changbo@hyit.edu.cn; 2 Faculty of Automation, HuaiYin Institute of Technology, Huaian 223003, China; 212409611039@hyit.edu.cn; 3 Key Laboratory of Thermo-Fluid Science & Engineering of MOE, Xi’an Jiaotong University, Xi’an 710049, China

**Keywords:** wireless sensor network, localization, time of arrival, Cramér–Rao lower bound, Kalman filtering

## Abstract

A two-stage fusion filtering positioning algorithm based on prediction residuals and gain adaptation is proposed to address the problems of disturbance and modeling errors in the application of distance-based positioning algorithms in wireless sensor networks, as well as inaccurate initial filtering values leading to large estimation errors or even divergence. Firstly, based on parameterization methods, a pseudo linear equation is constructed from the time of arrival (TOA) and multipath delay. The weighted least squares (WLS) method is applied to obtain the initial value of target position resolution, and its performance approaches the Cramér–Rao lower bound (CRLB). Secondly, the exact position of the target is obtained using the reconstructed Gaussian white noise statistics and the Kalman filtering algorithm. The simulation results show that compared with other initial positioning algorithms, the average positioning accuracy of the proposed algorithm is improved by 28.57%, and it has a better filtering performance.

## 1. Introduction

Wireless sensor networks (WSNs) have functions such as wireless communication and information collection and processing. They can combine wireless communication and network technology to perceive, transmit data, and provide terminal services for the target object in a monitoring environment [1,2]. The prominent feature of WSNs applied to the Internet of Things (IoT) architecture is the random broadcasting and distributed computing of a large number of nodes. It is an unattainable advantage of other detection means using sensor nodes carrying signal processing devices to implement information collection and conversion, especially to complete specific tasks in inaccessible military, civilian, and commercial areas, such as positioning and tracking [3,4]. In various engineering fields, the integration of data-driven approaches represented by artificial intelligence (AI) and optimization techniques has shown significant potential for improving system performance. For example, these techniques have been used in thermal engineering [5,6,7] and renewable energy consumption [8,9]. Similarly, in wireless sensor networks (WSNs), advanced optimization and AI techniques can enhance the accuracy and robustness of localization algorithms, improving overall performance. Nodes are at the very end of WSN applications, and the location information of nodes or targets is related to the application quality requirements of the scene and the implementation of monitoring networks. The techniques for implementing position estimation mainly include distance, time, and angle measurement, as well as the collection, organization, and processing of other crucial information that can participate in positioning. In terms of positioning quality, qualitative judgment and quantitative calculation of performance indicators such as computational complexity and real-time requirements should also be considered [10]. The two-dimensional positioning application scenario has led to extensive applications in various industrial fields, such as highways, medicine, public places, and emergency rescue. The current highly studied three-dimensional location estimation can fully exploit the potential of location perception and realize position-based advanced service functions, for which the classification and fusion technology of positioning has emerged [11,12].

In the study of positioning problems in WSN, according to whether distance is measured, the positioning algorithm can be divided into two kinds: ranging-based and non-ranging. Generally, the ranging-based positioning method has a higher accuracy. Common ranging methods such as TOA (time of arrival), AOA (angle of arrival), TDOA (time difference of arrival), FOA (frequency of arrival), and RSS (received signal strength) belong to the ranging-based techniques.

TOA technology is widely used in target positioning scenarios with high-performance requirements in indoor and outdoor areas due to its accurate distance measurement. A positioning network can estimate the location of massive nodes or targets by calculating the arrival time received by at least three sensor nodes, or implement precise positioning using collaborative techniques, ranging and inertial combination, and combined spatial–temporal arrays [13,14,15]. For example, Garraffa et al. [16] proposed a control-theoretic framework for range-inertial localization, rigorously analyzing error dynamics and convergence properties—a theoretical cornerstone for our filtering approach. Complementary to this, Feng et al. [17] demonstrated a Kalman filter-based fusion of UWB and IMU data, effectively suppressing observation noise and enhancing robustness in indoor navigation. In positioning methods based on distance measurement or angle measurement, such as AOA, RSS, and FOA, high-precision experimental results can be achieved if two or three of them are used for mixed positioning. In [18], the author studied the optimal sensor placement strategy for single static target positioning using mixed RSS, AOA, and TOA in a 2D plane, derived the theoretical minimum trace of the Cramér–Rao lower bound (CRLB) using the optimal criterion, proposed the corresponding optimal sensor placement strategy, and finally verified the results through simulation examples. In [19], the authors used TOA and RSS measurements to accurately locate the target in a non-line of sight (NLOS) environment, enhancing positioning accuracy. Starting from the general approximation of the maximum likelihood (ML) estimation problem, the authors established the nonlinear weighted least squares (NLWLS) using the equilibrium parameter, then used semi-definite relaxation (SDR) technology to solve the NLWLS problem, and the numerical simulation proved that the proposed method significantly outperforms the existing methods.

To improve the positioning accuracy of the WSN network, Kalman filtering and particle filtering methods can also be used. The Kalman filtering is an efficient recursive filter capable of estimating the states of dynamic systems from a series of incomplete and noisy inclusive measurements and is particularly suitable for handling linear systems and systems with Gaussian white noise. Particle filtering can deal with nonlinear, non-Gaussian localization problems caused by environmental complexity (such as multipath effect, NLOS propagation). In practical applications, the two methods can also be combined to further improve positioning accuracy and robustness. The development of Kalman filtering series algorithms has gone through several stages, from the initial Kalman filter (KF) to the extended Kalman filter (EKF) for nonlinear systems, to the untracked Kalman filter (UKF), these algorithms have played an important role in their respective application fields.

Although the particle filter series algorithm is effective when dealing with nonlinear systems, the particle filter has the disadvantages of degradation and a large amount of computation, making it difficult to the strict requirements of limited positioning resources in WSN. Therefore, the Kalman filter series algorithm is the better method to deal with the positioning problem of nonlinear systems based on ranging [20,21]. The series algorithms of Kalman filtering have developed rapidly in recent years and have been applied in various data and information processing scenarios [22,23]. In the field of positioning, tracking, and navigation based on ranging technology, it is often seen that Kalman filtering has a superior performance in accurate estimation, which is used to reduce the process or measurement error, improve the positioning accuracy, and obtain satisfactory positioning tracking and navigation effects [24,25,26,27]. UKF is a filtering method for the nonlinear system and its core is the differential-free suboptimal algorithm of unscented transformation (UT), that is, from approaching a nonlinear function to approaching a probability distribution, so that the calculation process is optimized and the filtering effect is significantly improved. Therefore, many application scenarios have been developed [28,29,30,31].

Many factors in the system positioning process adversely affect the location estimation, and this paper mainly considers the decrease in localization accuracy caused by ranging error and colored measurement noise. Based on the above requirements of ranging problems and real-time positioning, this study mainly makes the following contributions:

- Setting the distance measurement based on TOA as a Gaussian stochastic process, adopting the nonlinear system parametric method to derive the pseudo-linear equation with unknown multipath delay, then applying the weighted least squares (WLS) criterion and the effective analytical calculation of process parameters to obtain the approximate solution for the target primary position estimation that can approximate the CRLB based on TOA measurement estimation.
- Realizing the optimal estimation, using the new Gaussian distributed white noise statistic to whiten the observed colored noise to eliminate the adverse effect of the colored noise on the filter localization, and update the Kalman filter parameters after obtaining the new measurement model.
- Setting the correlation coupling matrix to realize the decoupling according to the independent conditions of process and observation error, to derive the filter decoupling measurement model, and then update the filter parameters.
- Because of the problem of error in noise modeling, realizing self-optimization filtering estimation using predicting residual statistics, noise covariance, and gain update, and by compensating the estimation error again to accelerate localization convergence and enhance the robustness of the system. Finally, the feasibility and effectiveness of the positioning algorithm are verified by numerical simulation experiments.

## 2. Related Work

The scenario based on location information mainly appears in various application fields, and location estimation can make full use of the potential of position perception and realize location-based advanced service functions, so the technology and algorithm development on positioning have shown an amazing development speed. In [32], the authors proposed a robust localization algorithm based on a Gaussian mixture model and fitting polynomials for the positioning error problem in complex indoor environments. Firstly, the predicted and measured residues are clustered using the Gaussian mixture model (GMM) and the line of sight (LOS) probability and non-line of sight (NLOS) probability are calculated; then, the measured values are filtered through the Kalman filter (KF), variable parameter unscented Kalman filter (VPUKF), and variable parameter particle filter (VPPF); finally, position coordinate estimates are calculated using the maximum likelihood method. The simulation results showed that the proposed algorithm has a better localization accuracy and robustness than several other comparison algorithms. In [33], the authors proposed a new robust matrix approximation scheme using multidimensional similarity (MDS) analysis under non-line of sight (NLOS) propagation and applied the multiplier’s alternating direction method to solve the resulting nonlinear constraint optimization problem. The simulation results showed that the proposed method is superior to several existing methods. In the ranging positioning technology based on TOA, the time synchronization problem is relatively prominent, and when the asynchrony is serious, the positioning error will be too large or even result in positioning failure. Therefore, it is necessary to put forward the positioning algorithm to consider the synchronization problem [34]. In [35], the authors analyzed the strict time synchronization between the source and the base station required for TOA localization, and introduced the synchronization error into the TOA-based NLOS localization model, considering that NLOS propagation significantly reduces positioning precision.

Similarly, to solve the asynchronous positioning problem, in [36], considering the positioning and tracking in the application of autonomous underwater vehicles (AUVs), the authors first developed an asynchronous positioning algorithm to estimate the location of AUVs and designed a model-free tracking controller for AUVs to track the reference trajectory. In addition, they derived the convergence conditions and CRLB of the localization algorithm, constructed the Lyapunov function to analyze its stability, and verified its effectiveness through simulation experiments, using an optimization scheme to achieve high-precision positioning is an effective way to further reduce position errors. In [37], the authors studied the localization of multiple signal sources, proposed a self-clustering measurement combination method, using the source location estimation obtained by hyperbolic positioning algorithm as the clustering mode, defined the scatter of the patterns of different subsets of TOA measurements as the criterion function, adopted the three-step heuristic clustering algorithm to solve the TOA ambiguity, and analyzed the mean square error performance and computational complexity. In [38], the authors applied collaborative technology to achieve positioning and proposed a non-convex robust weighted least squares (RWLS) problem to locate multiple stationary target nodes. With the help of semi-definite relaxation technology, the RWLS problem is transformed into a convex semi-definite second-order cone hybrid programming problem, and both the simulation and actual experimental data verified the superior performance of the proposed method. Back to the positioning model itself, in [39], based on the single mobile station positioning system, according to the available measurement, including AOA, TOA, and FOA, the authors derived the corresponding pseudo linear equation, established the total least squares optimization model, and used the inverse iterative algorithm to solve the static target position with a theoretical derivation and rigorous proof to achieve CRLB performance.

The positioning algorithms based on single ranging or joint ranging listed above solve some problems from the perspective of different application scenarios, have certain applicability in specific environments, and provide ideas for future positioning exploration. With the change in scene time and space, it is still necessary to develop new technologies for different positioning environments and constantly innovate and improve them.

Kalman filtering methods have been widely applied in data and information processing scenarios of various dynamic systems. Kalman filtering in the field of positioning, tracking, and navigation has a superior performance and is used to reduce process or measurement error and improve positioning accuracy. The study of Gaussian white noise is relatively mature, but colored noise is widespread in actual localization systems, so it is necessary to study the latter [40]. One of the biggest challenges encountered in applying Kalman filtering in location-tracking scenarios based on ranging technology is the noise processing problem at the measurement end. This problem often shows us how to deal with the colored noise to make it white, to be suitable for Kalman filtering, and to obtain improved positioning accuracy and good robustness [41,42]. In [41], since the existence of colored noise in global navigation satellite system (GNSS) measurement will reduce the performance of fault detection and exclusion (FDE) algorithm based on KF, the authors proposed an FDE scheme based on improved KF, using the colored noise as a first-order self-regression model to improve the performance of FDE, and evaluated the performance of the proposed algorithm through real-time kinematic positioning application, obtaining good positioning results. In [42], considering state coupling and colored noise among system nodes, the authors designed a robust extended state Kalman filter estimation algorithm for nonlinear complex networks. The innovative measurement information subtraction method at adjacent moments deals with colored noise, which greatly reduces the influence of network bandwidth constraints, obtains upper bounds of error covariance, and achieves real-time gain optimization. The rationality and accuracy of the proposed estimator are verified by simulation.

It can be seen from the above studies that some effective methods have been developed for the problem of reducing the algorithm performance in the measurement process contaminated by colored noise. However, most algorithms still have the problem of the processed white noise sequence involving the coupling between the system process and the observation process. In addition, some problems such as the cumbersome algorithm design process and the increasing estimation cost with the sampling frequency are prominent. Therefore, the robustness and efficiency of the algorithm should be the focus of the next consideration.

UKF propagates statistical properties via no-trace nonlinear transformations and employs weighted statistical linear regression models to calculate the mean and covariance of random variables to obtain updates of the state estimates, especially suitable for target localization based on nonlinear ranging scenarios. In [43], the authors consider that the choice of process noise covariance matrix and measurement noise covariance matrix will directly affect the filtering accuracy of the algorithm, and propose using a hybrid algorithm including UKF and the genetic particle swarm algorithm to estimate several key states of the vehicle. The simulation and real vehicle test results show that the algorithm has a higher accuracy and less computational complexity than the traditional estimators based on UKF and traceless particle filters. In [44], the authors extended the special requirements for positioning application scenarios, designed and developed a positioning framework composed of three algorithms, proposed an improved Kalman filter positioning method based on UKF and PF positioning, and verified the robustness of the proposed algorithm through numerical simulation. The simulation results show that the proposed positioning algorithm can be used for target tracking, robot positioning, and other purposes.

The above UKF-based filtering improvement algorithm or the combined filtering positioning algorithm have achieved a good application effect in different positioning environments. However, some algorithms only provide beneficial exploration, but it is difficult to achieve in practice. With the change in scene time and space, it is still necessary to develop new technologies to continuously innovate and improve from the aspects of improving performance and stronger adaptability.

This work proposes a two-stage fusion filter positioning algorithm, which can be quickly converged based on the original algorithm performance and achieve the purpose of optimizing the performance. The techniques used in this paper include Gaussian noise modeling, the analytical method of weighted least squares criterion, derivation of the Cramér–Rao lower bound, colored noise signal processing, multi-source information fusion, and gain adaptive filtering, etc. Comprehensive application of these technologies in target positioning can obtain the precise position of the target, enhance the robustness of the system, and obtain a better positioning effect. The experimental results also demonstrate that the fusion algorithm is consistent with the theoretical derivation, the average localization accuracy improves, and the system has a better accuracy and filtering stability.

## 3. TOA Ranging Modeling and Initial Positioning

### 3.1. Location Information Modeling

If TOA ranging is used for positioning, assuming that the electromagnetic pulse is emitted at time *t*_0_, the arrival time of the receiving pulse at time *t_i_*, $t_{i}$ can be expressed as follows.

(1) $$t_{i}=t_{0}+\frac{D_{i}}{c}$$

where $D_{i}$ is the distance between the transmitter and the receiver, and *c* is the light speed.

Considering the influence of path delay in the wireless signal transmission process, the transmission mode between any two points on the signal path is nonlinear propagation, which is usually processed mathematically as propagation along a straight line, thus forming a ranging error. Therefore, considering the integrated path delay influence, the wireless signal path observation equation based on TOA is as follows:

(2) $$t_{i}=t_{0}+t_{y}+\frac{D_{i}}{c}$$

where $t_{y}$ is the channel integrated path delay.

In this work, the 3D case is considered, where the position of the target node or the anchor node is represented by a column vector consisting of three coordinates. Let the set consisting of N target nodes be $\Psi_{m}=\left\{m_{1},m_{2},\cdots ,m_{N}\right\}$, randomly or consciously deployed in a three-dimensional region. The true position of the target is represented as $z={\left[z_{x},\;z_{y}{,z}_{z}\right]}^{T}$, where *z* is unknown and its estimated position is represented as $\hat{z}={\left[{\hat{z}}_{x},{\hat{z}}_{y},{\hat{z}}_{z}\right]}^{T}$. Moreover, assuming that M anchor nodes are already equipped with GPS or have obtained precise position by the positioning algorithm, the position of the anchor nodes can be expressed as $m={\left[m_{x},m_{y},m_{z}\right]}^{T}$, and M anchor nodes can be expressed as $\Psi_{a}=\left\{a_{1},a_{2},\cdots ,a_{M}\right\}$. Generally, there is M≪N, and there are at least 4 anchor nodes within the communication range of each target node.

The TOA-based measured distances between the target and the anchor nodes are modeled as

(3) $$R_{ik}=c\left(t_{ik}-t_{0}-t_{y}\right)=\left\|z_{i}-m_{k}\right\|+v_{ik}$$

where ‖∗‖ is the norm operator, and $v_{ik}$ $\left(k=1,\;2,\;\cdots ,\;M\right)$, modeled as the corresponding measurement noise, is a random variable following an independent Gaussian homogeneous distribution with a mean of zero.

The location of target nodes can be determined using target positioning technology and obtained as accurately as possible using $R_{ik}$ with noise. In this paper, we consider using the measurement information to implement the simultaneous estimation of $z_{i}$ and $t_{y}$.

### 3.2. Positioning Algorithm Based on the Observed Pseudo-Linearized Analytical Solution

Based on the TOA measurement equation shown in Equation (3), assuming that the estimates of $z_{i}$ and $t_{y}$ are ${\hat{z}}_{i}$ and ${\hat{t}}_{y}$, respectively, the square of Equation (3) is treated and adjusted, and set ${\left(c{\hat{t}}_{y}\right)}^{2}-{\left\|{\hat{z}}_{i}\right\|}^{2}=\varrho$, then

(4) $${\left(ct_{ik}-{ct}_{0}\right)}^{2}-{\left\|m_{k}\right\|}^{2}+\varrho =2\left(ct_{ik}-{ct}_{0}\right)c{\hat{t}}_{y}-2{m_{k}}^{T}{\hat{z}}_{i}+2\left(ct_{ik}-{ct}_{0}-c{\hat{t}}_{y}\right)v_{ik}-{v_{ik}}^{2}$$

The matrix form of Equation (4) is given by

(5) $$A_{c}+1\varrho =A_{t}\theta +V_{v}$$

where

$$A_{c}=\left[\begin{array}{c} {\left(ct_{i1}-{ct}_{0}\right)}^{2}-{\left\|m_{1}\right\|}^{2}\\ \vdots \\ {\left(ct_{M}-{ct}_{0}\right)}^{2}-{\left\|m_{M}\right\|}^{2} \end{array}\right],\;1=\left[\begin{array}{c} 1\\ \vdots \\ 1 \end{array}\right],\;A_{t}=\left[\begin{array}{c} 2c\left(ct_{i1}-{ct}_{0}\right)-2{m_{1}}^{T}\\ \vdots \\ 2c\left(ct_{M}-{ct}_{0}\right)-2{m_{M}}^{T} \end{array}\right],\;V_{v}=\left[\begin{array}{c} 2\left(ct_{i1}-{ct}_{0}-c{\hat{t}}_{y}\right)v_{i1}-{v_{i1}}^{2}\\ \vdots \\ 2\left(ct_{iM}-{ct}_{0}-c{\hat{t}}_{y}\right)v_{iM}-{v_{iM}}^{2} \end{array}\right]$$

$\theta ={\left[\begin{array}{cc} {\hat{t}}_{y} & {{\hat{z}}_{i}}^{T} \end{array}\right]}^{T}$ is the unknown vector to be estimated, and $V_{v}$ is the noise vector.

For non-Gaussian noise $V_{v}$, assuming its variance is $\sigma_{n}^{2}$, by setting a vector $R_{v}$ and using ${R_{v}}^{-1/2}$ to achieve non Gaussian noise processing, that is, by calculating ${R_{v}}^{-1/2}V_{v}$ and obtaining standard Gaussian noise, it should be multiplied ${R_{v}}^{-1/2}$ at both ends of the Equation (5). This is a general process for handling non Gaussian noise.

Assuming $E\left[V_{v}{V_{v}}^{T}\right]/\sigma_{n}^{2}=R_{v}$, non-Gaussian noise is processed into Gaussian noise, yielding

(6) $${R_{v}}^{-1/2}A_{c}+{R_{v}}^{-1/2}1\varrho ={R_{v}}^{-1/2}A_{t}\theta +{R_{v}}^{-1/2}V_{v}$$

Thus, the estimate of *θ* is

(7) $$\hat{\theta }=\alpha +\beta \varrho$$

where

$${\alpha =\left({A_{t}}^{T}{R_{v}}^{-1}A_{t}\right)}^{-1}{A_{t}}^{T}{R_{v}}^{-1}A_{c}\;\beta ={\left({A_{t}}^{T}{R_{v}}^{-1}A_{t}\right)}^{-1}{A_{t}}^{T}{R_{v}}^{-1}\;\;\;\;\;$$

then

$$R_{v}=E\left[V_{v}{V_{v}}^{T}\right]/\sigma_{n}^{2}=\left[\begin{array}{ccc} 4{\left\|{\hat{z}}_{i}-m_{1}\right\|}^{2}+{3\sigma }_{n}^{2} & \cdots & \sigma_{n}^{2}\\ \vdots & \ddots & \vdots \\ \sigma_{n}^{2} & \cdots & 4{\left\|{\hat{z}}_{i}-m_{M}\right\|}^{2}+{3\sigma }_{n}^{2} \end{array}\right]$$

The initial value of $R_{v}$ is usually set to be the unit matrix *I*, and weighted least squares are applied for the $R_{v}$. There is usually no prior value for $\sigma_{n}^{2}$ in the actual localization, and in subsequent calculations, $R_{v}$ is set to

(8) $$R_{v}\approx diag\left\{{4\left(ct_{i1}-{ct}_{0}-c{\hat{t}}_{y}\right)}^{2},\cdots ,{4\left(ct_{iM}-{ct}_{0}-c{\hat{t}}_{y}\right)}^{2}\right\}$$

Set the estimate of *ϱ* as $\hat{\varrho }$, then

(9) $$\beta^{T}Bb{\hat{\varrho }}^{2}+\left(2\beta^{T}B\alpha -1\right)\hat{\varrho }+\alpha^{T}Q\alpha =0$$

where $B=\left[\begin{array}{ccc} 1 & 0 & 0\\ 0 & -1 & 0\\ 0 & 0 & -1 \end{array}\right]$. Therefore, we obtain the $\hat{\varrho }$ by analytical solution and can then compute $\hat{\theta }$. Register ${\left(2\beta^{T}B\alpha -1\right)}^{2}-4\beta^{T}B\beta \alpha^{T}Q\alpha =\gamma$; then, for the case of $\beta^{T}B\beta =0$, the solution of $\hat{\varrho }$ is

$$\hat{\varrho }=\frac{\alpha^{T}B\alpha }{1-2\beta^{T}Q\alpha }$$

Thus, the localization solution is

(10) $$\hat{\theta }=\left[\begin{array}{c} {\hat{t}}_{y}\\ {\hat{z}}_{i} \end{array}\right]=\alpha +\beta \frac{\alpha^{T}B\alpha }{1-2\beta^{T}B\alpha }$$

If $\beta^{T}B\beta \neq 0$ and γ=0, there is a unique solution of $\hat{\varrho }$, and the localization solution is

(11) $$\hat{\theta }=\left[\begin{array}{c} {\hat{t}}_{y}\\ {\hat{z}}_{i} \end{array}\right]=\alpha +\beta \frac{1-2\beta^{T}B\alpha }{2\beta^{T}B\beta }$$

If $\beta^{T}B\beta \neq 0$ and γ>0, there are two different solutions of $\hat{\varrho }$, and the localization solution is

(12) $$\hat{\theta }=\left[\begin{array}{c} {\hat{t}}_{y}\\ {\hat{z}}_{i} \end{array}\right]=\alpha +\beta \frac{1-2\beta^{T}B\alpha \pm \sqrt{{\left(2\beta^{T}B\alpha -1\right)}^{2}-4\beta^{T}B\beta \alpha^{T}B\alpha }}{2\beta^{T}B\beta }$$

Solving the uniqueness problem of the localization solution can be obtained using the least squares criterion based on the TOA measurement.

(13) $$\underset{{\hat{t}}_{y},{\hat{z}}_{i}}{\min}\sum_{k=1}^{M}{\left(\left\|{\hat{z}}_{i}-m_{k}\right\|-ct_{ik}+{ct}_{0}+c{\hat{t}}_{y}\right)}^{2}$$

In other cases, there is no effective solution, and at this time, the effective measurement value cannot be obtained, so other alternative algorithms can be sought to solve it. The details of the initial localization algorithm steps are illustrated in Algorithm 1.
**Algorithm 1.** The initial localization algorithm1: Initialization, set R, calculate α, β, and ϱ.2: If γ<0, then select the other algorithm as an alternative.3: If $\beta^{T}B\beta =0$, then have $\hat{\theta }$ from Equation (10).4: If γ=0, then have $\hat{\theta }$ from Equation (11).5: If γ>0, then obtain two $\hat{\theta }$ from Equation (12).6: the uniqueness of the solution can be solved by Equation (13).7: return.

## 4. Self-Optimized Target Filter Localization Method with Colour Noise Observation

In terms of processing strong nonlinear systems such as target positioning systems, the significant advantages of the unscented Kalman filter (UKF) algorithm are filtering accuracy, algorithm stability, and computational load. As with the basic linear filtering method, for the UKF algorithm, we also assume that the process noise and observation noise are Gaussian white noise. However, in the location tracking application, influenced by the environment and facilities, the algorithm model will include colored noise characteristics, which may usually affect the filter accuracy or directly lead to filter divergence.

### 4.1. Filter Localization Problem Modeling and the Basic UKF Algorithm

#### 4.1.1. Filter Localization Problem Modeling

If a target moves in a constant straight line in a short period, the acceleration is theoretically 0. But in practice, the acceleration of the target affected by itself and the environment is abstracted as a random variable, which can be modeled as a zero-mean Gaussian white noise process at time *t*, namely $\ddot{x}=∁\left(t\right)$, whose mean is $E\left[∁\left(t\right)\right]=0$ and covariance is $E\left[∁\left(t\right)∁\left(\tau \right)\right]=q\left(t\right)\delta \left(t-\tau \right)$, where $\delta \left(t-\tau \right)=0$ at t≠τ and $\delta \left(t-\tau \right)=1$ at t=τ; that is, the white noise is only correlated with its current moment.

Suppose that the position coordinates and speed of the target in the 3-D system are $\left(x,y,z\right)$ and $\left(v_{x},v_{y},v_{z}\right)$, respectively, and its state vector can be expressed as $X=[X_{p},X_{v}]$, where *X* represents the state vector of the positioning system, $X_{p}={\left[\begin{array}{ccc} x & y & z \end{array}\right]}^{T}$, and $X_{v}={\left[\begin{array}{ccc} v_{x} & v_{y} & v_{z} \end{array}\right]}^{T}$. Then, the continuous-time equation of the state of the system is expressed as follows:

(14) $$\dot{X}\left(t\right)=AX\left(t\right)+BW\left(t\right)$$

where *A* represents the state transition matrix, *B* is the process noise allocation matrix, and *W*(*t*) represents the process noise, which is a mutually independent white noise vector component with a zero-mean Gaussian distribution. To facilitate digital filtering estimation, the discretized equation of state is set as

(15) $$X_{k+1}=f\left(X_{k}\right)+W_{k}=FX_{k}+BW_{k}$$

where $F=\left[\begin{array}{cc} 1_{3\times 3} & T_{3\times 3}\\ O_{3\times 3} & 1_{3\times 3} \end{array}\right]$ represents the state transition matrix from discrete time *k* to *k* + 1; *k* represents the discrete time; *T* represents the sampling time; $X_{k+1}$ represents the state vector of the system at the discrete time *k* + 1; *B* represents the process noise allocation matrix from discrete time *k* to *k* + 1; and $W_{k}$ represents the process noise at discrete time *k*, which satisfies $E\left[W_{k}\right]=0$ and $cov\left[{W_{k}W}_{j}\right]=Q_{k}\delta_{kj}$.

The measuring device can obtain the distance information from the anchor node to the target. For the TOA of the received signal at the anchor node, the distance was calculated using the equation $d=cT=\left\|z_{i}-m_{k}\right\|$. Thus, the distance measurements can be modeled as

(16) $$d=h\left(x\right)=cT=\left\|z_{i}-m_{k}\right\|$$

where $h\left(x\right)$ is a nonlinear function of the calculated distance information. Considering the observation error of the measurement equipment, the following observation equation can be established

(17) $$Z_{k}=h\left(X_{k}\right)+V_{k}$$

where $f\left(X_{k}\right)$ is the vector of nonlinear observation functions, and $V_{k}$ is the observation noise vector of the measurement device.

#### 4.1.2. The Basic UKF Algorithm

The unscented transformation (UT) can directly find the mean and covariance of the target distribution, avoiding the approximation of nonlinear function, so the unscented filtering effect is next only to particle filtering, but the computational load is less, which is more suitable for low-dimensional strong nonlinear systems such as ranging positioning.

For KF class filtering, we consider only the additive noise, namely, both $W_{k}$ and $V_{k}$ are zero-mean Gaussian white noise. The process noise variance of the positioning system is

(18) $$\;\;\;\;E\left[\left(B_{k}W_{k}\right){\left(B_{k}W_{k}\right)}^{T}\right]=E\left[\left(B_{k}\left(W_{k}W_{k}^{T}\right)B_{k}^{T}\right)\right]=E\left[\left(B_{k}{℧}_{k}B_{j}^{T}\right)\right]=Q_{k}\delta_{kj}$$

where $\delta_{j}$ is the Kronecker−δ function, taking 1 if k=j and 0 if k≠j. The variance of the observed noise $V_{k}$ is $R_{k}$, which satisfies $E\left[\left(V_{k}V_{j}^{T}\right)\right]=R_{k}\delta_{kj}$, and assumes that $W_{k}$ is not correlated with $V_{k}$.

In this study, a scaled UT transformation method called SUT transformation is adopted to give full play to the filtering performance. The sigma points $\chi_{k-1}^{0},\chi_{k-1}^{1},\cdots ,\chi_{k-1}^{2n}$ are selected according to the selection rule using Equation (19).

(19) $$\left\{\begin{array}{c} \chi^{0}=\bar{x},\\ \chi^{i}=\bar{x}+\sqrt{n+\lambda }{\left[\sqrt{P_{x}}\right]}_{i}\\ \chi^{n+i}=\bar{x}-\sqrt{n+\lambda }{\left[\sqrt{P_{x}}\right]}_{i}, \end{array}\right.,i=1,\cdots ,n$$

Using Equation (20) to determine the weight value $w_{0},w_{1},\cdots ,w_{2n}$,

(20) $$\left\{\begin{array}{c} w_{0}^{\left(m\right)}=\lambda /\left(n+\lambda \right),\\ w_{0}^{\left(c\right)}=\lambda /\left(n+\lambda \right)+(1-\alpha^{2}+\beta )\\ w_{i}^{\left(m\right)}=w_{i}^{\left(c\right)}=1/\left(2n+2\lambda \right), \end{array}\right.,i=1,\cdots ,2n$$

where $\lambda =\alpha^{2}(n+\kappa )-n$. We set the value range of parameter α as $10^{-4}\leq \alpha <1$, κ we usually select as 0 (state dimension N≥3) or 3−n (state dimension N<3), and we select *β* = 2 when the noise is a Gaussian distribution. ${\left[\cdot \right]}_{i}$ represents column *i* of the matrix, and each sigma point constitutes each column of the sigma matrix.

The computational flow of the basic UKF algorithm is described as follows:

(1) Initialization

Step 1: Initialize the state ${\hat{X}}_{0}$ and its covariance $P_{0}$, $Q_{0}$, and $R_{0}$; ${\hat{X}}_{0}$ is the state estimation vector at the initial time (time 0); $P_{0}$ is the state covariance matrix at the initial time, $Q_{0}$ is the initial process noise variance matrix; and $R_{0}$ is the initial observation noise variance matrix. For a series of moments *k*, the loop proceeds from the algorithm Step (2) to Step (6).

(2) Prediction

Step 2: The sigma point nonlinear transformation is performed according to the estimated mean ${\hat{X}}_{k-1}$, the covariance matrix $P_{k-1}$, and the system dynamic model, that is

(21) $${\hat{\chi }}_{\left.k\right|k-1}^{i}=f\left(\chi_{k-1}^{i}\right),\;i=0,\cdots ,2n$$

Step 3: For the state mean and covariance, the predicted values are given by Equations (22) and (23), respectively.

(22) $${\hat{X}}_{\left.k\right|k-1}=\sum_{i=0}^{2n}w_{i}^{\left(m\right)}{\hat{\chi }}_{\left.k\right|k-1}^{i}$$

(23) $$P_{\left.k\right|k-1}=\sum_{i=0}^{2n}w_{i}^{\left(c\right)}\left({\hat{\chi }}_{\left.k\right|k-1}^{i}-{\hat{X}}_{\left.k\right|k-1}\right){\left({\hat{\chi }}_{\left.k\right|k-1}^{i}-{\hat{X}}_{\left.k\right|k-1}\right)}^{T}+Q_{k-1}\;$$

(3) Updating

Step 4: The sigma point nonlinear transformation is performed according to the measurement model, i.e.,

(24) $${\hat{Z}}_{\left.k\right|k-1}^{i}=h\left({\hat{\chi }}_{\left.k\right|k-1}^{i}\right),\;i=0,\cdots ,2n$$

Step 5: Measurement predicts the mean, innovation covariance, mutual covariance between state and measurement, and filter gain as follows:

(25) $${\hat{Z}}_{\left.k\right|k-1}=\sum_{i=0}^{2n}w_{i}^{\left(m\right)}{\hat{Z}}_{\left.k\right|k-1}^{i}$$

(26) $$S_{k}=\sum_{i=0}^{2n}w_{i}^{\left(c\right)}\left({\hat{Z}}_{\left.k\right|k-1}^{i}-{\hat{Z}}_{\left.k\right|k-1}\right){\left({\hat{Z}}_{\left.k\right|k-1}^{i}-{\hat{Z}}_{\left.k\right|k-1}\right)}^{T}+R_{k}$$

(27) $$C_{k}=\sum_{i=0}^{2n}w_{i}^{\left(c\right)}\left({\hat{\chi }}_{\left.k\right|k-1}^{i}-{\hat{X}}_{\left.k\right|k-1}\right){\left({\hat{Z}}_{\left.k\right|k-1}^{i}-{\hat{Z}}_{\left.k\right|k-1}\right)}^{T}\;$$

(28) $$K_{k}=C_{k}S_{k}^{-1}$$

Step 6: The mean and covariance matrix of the posterior state estimation at time *k* are given by Equations (29) and (30), respectively.

(29) $${\hat{X}}_{k}={\hat{X}}_{\left.k\right|k-1}+K_{k}\left(Z_{k}-{\hat{Z}}_{\left.k\right|k-1}\right)$$

(30) $$P_{k}=P_{\left.k\right|k-1}-K_{k}S_{k}K_{k}^{T}$$

### 4.2. Nonlinear Filtering of the Localization System Based on the Whitening of the Colored Noise

#### 4.2.1. Colored Noise Modeling and Whitening-Based Filtering Methods

The observed noise based on TOA measurement mainly refers to the colored noise, which can achieve the optimal estimation performance under the framework of the KF algorithm. Using a time-series analysis model, a smooth colored noise sequence can be composed of related sequences and white noise at each time, which is called an autoregressive sliding average model ARMA (p, q). In this paper, q = 0, p = 1, and colored noise are modeled as in the AR (1) model.

The output of Gaussian distributed white noise after passing the first-order linear filter is the colored noise $V_{k}$, which can be expressed as

(31) $$V_{k}=ðV_{k-1}+\nu_{k}$$

where ð=1−T/τ, $\nu_{k}=(T/\tau )\theta_{k}$, $\nu_{k}$ is the Gaussian white noise with a mean of 0 and a variance of ${R_{k}=R}_{r}{\left(T/\tau \right)}^{2}$, where $R_{r}$ is the variance of the Gaussian distribution $\theta_{k}$.

$V_{k}$ in the system observation model is a colored noise vector, which is whitened so that the noise condition meets the UKF application requirements to obtain a good performance.

Consider the discretized system states and the observed model as follows:

(32) $$X_{k}=F_{k-1}X_{k-1}+B_{k-1}W_{k-1}$$

(33) $$Z_{k}=H_{k}X_{k}+V_{k}$$

Then, the colored noise $V_{k}=Z_{k}-H_{k}X_{k}$; considering the colored noise $V_{k-1}$, the corresponding observation model is as follows:

(34) $$Z_{k-1}=H_{k-1}X_{k-1}+V_{k-1}\;\to V_{k-1}=Z_{k-1}-H_{k-1}X_{k-1}$$

Then,

(35) $${ðV}_{k-1}={ðZ}_{k-1}-{ðH}_{k-1}X_{k-1}$$

According to Equations (32)–(35), $\left(V_{k}-ðV_{k-1}\right)$-type Gaussian distribution white noise is reconstructed, and we can obtain the following:

(36) $$\begin{array}{cc} v_{k}\; & {=V}_{k}-ðV_{k-1}\\ & =Z_{k}-H_{k}X_{k}-{ðZ}_{k-1}-{ðH}_{k-1}X_{k-1}\\ & =Z_{k}-ðZ_{k-1}-X_{k-1}\left(H_{k}F_{k-1}-ðH_{k-1}\right)\\ & +H_{k}B_{k-1}W_{k-1} \end{array}$$

Equation (36) can be abbreviated as

(37) $$Z_{k}^{z}=H_{k}^{h}X_{k}+V_{k}^{v}$$

where

$$Z_{k}^{z}=Z_{k}-ðZ_{k-1}\;H_{k}^{h}=H_{k}F_{k-1}-ðH_{k-1}\;V_{k}^{v}=H_{k}B_{k-1}W_{k-1}+v_{k}$$

Here, $V_{k}^{v}$ is the new noise, and its mean value is

(38) $$E\left[V_{k}^{v}\right]=E\left[H_{k}B_{k-1}W_{k-1}+v_{k}\right]=0$$

That is, $V_{k}^{v}$ is the white noise with a mean of 0, which realizes the colored noise whitening processing.

The variance of $V_{k}^{v}$ is

(39) $$\begin{array}{cc} & E\left[V_{k}^{v}{V_{k}^{v}}^{T}\right]\\ =\; & E\left[\left(H_{k}B_{k-1}W_{k-1}+v_{k}\right){\left(H_{j}B_{j-1}W_{j-1}+v_{j}\right)}^{T}\right]\\ =\; & \delta_{kj}\left(H_{k}Q_{k-1}H_{j}^{T}+R_{k}\right) \end{array}$$

Therefore, after the whitening of the colored noise, the filtering parameters can be updated as shown in Equations (40)–(43).

(40) $${\hat{\chi }}_{\left.k\right|k-1}^{i}=F_{k-1}\chi_{k-1}^{i},\;i=0,\cdots ,2n$$

(41) $${\hat{Z^{z}}}_{\left.k\right|k-1}^{i}=h\left({\hat{\chi }}_{\left.k\right|k-1}^{i}\right)=H_{k}^{h}\chi_{k-1}^{i},\;i=0,\cdots ,2n$$

(42) $${\hat{Z}}_{\left.k\right|k-1}=\sum_{i=0}^{2n}w_{i}^{\left(m\right)}{\hat{Z^{z}}}_{\left.k\right|k-1}^{i}$$

(43) $$S_{k}=\sum_{i=0}^{2n}w_{i}^{\left(c\right)}\left({\hat{Z^{z}}}_{\left.k\right|k-1}^{i}-{\hat{Z}}_{\left.k\right|k-1}\right){\left({\hat{Z^{z}}}_{\left.k\right|k-1}^{i}-{\hat{Z}}_{\left.k\right|k-1}\right)}^{T}+R_{k}^{r}$$

Here,

(44) $$R_{k}^{r}=H_{k}Q_{k-1}H_{j}^{T}+R_{k}$$

#### 4.2.2. Noise Decoupling Filtering Algorithm

Since UKF is based on the framework of the KF filter, after the whitening of the colored observation noise, the covariance of the process noise $W_{k}$ and the observation noise $V_{k}^{v}$ is $E\left[W_{k}{V_{k}^{v}}^{T}\right]=E\left[W_{k}{\left(H_{k}B_{k}W_{k}+v_{k}\right)}^{T}\right]\neq 0$, and there is a coupling between the observation noise and system noise, which needs to be decoupled to continue Kalman filtering. After the whitening of the colored observation noise, the discretized system state and the observation model are shown in Equations (45) and (46).

(45) $$X_{k}=F_{k-1}X_{k-1}+B_{k-1}W_{k-1}$$

(46) $$Z_{k}^{z}=H_{k}^{h}X_{k-1}+V_{k}^{v}$$

For the noise coupling of the state and the observation, the new observation model is set as

(47) $${\overset{˘}{Z}}_{k}^{z}=Z_{k}^{z}+{\overset{˘}{V}}_{k}^{v}$$

where $Z_{k}^{z}=H_{k}^{h}X_{k-1}+V_{k}^{v}$.

Setting the noise coupling matrix of the state and the observation be $O_{k-1}$, then the system state is expressed as

(48) $$X_{k}=F_{k-1}X_{k-1}+B_{k-1}W_{k-1}+O_{k-1}{\overset{˘}{V}}_{k}^{v}$$

and there is

(49) $$Z_{k}^{z}=H_{k}^{h}X_{k-1}+V_{k}^{v}\;\;$$

(50) $$X_{k}=F_{k-1}X_{k-1}+B_{k-1}W_{k-1}+O_{k-1}{\overset{˘}{V}}_{k}^{v}$$

For the system process and observation model, Equations (48)–(50) are combined, and we can obtain Equation (51).

(51) $$X_{k}=F_{k-1}^{f}X_{k-1}+O_{k-1}Z_{k}^{z}+{Ε}_{k}^{e}$$

where

$$F_{k-1}^{f}=\left(F_{k-1}-O_{k-1}H_{k}^{h}\right)$$

$${Ε}_{k}^{e}=B_{k-1}W_{k-1}-O_{k-1}V_{k}^{v}$$

The covariance matrix of the process error ${Ε}_{k}^{e}$ and the observation error $V_{k}^{v}$ is given as follows:

(52) $$E\left[\left(B_{k-1}W_{k-1}-O_{k-1}V_{k}^{v}\right){V_{k}^{v}}^{T}\right]\;=E\left[B_{k-1}W_{k-1}{V_{k}^{v}}^{T}\right]-E\left[G_{k-1}V_{k}^{v}{V_{k}^{v}}^{T}\right]\;\;\;\;=E\left[B_{k-1}W_{k-1}{V_{k}^{v}}^{T}\right]-G_{k-1}E\left[V_{k}^{v}{V_{k}^{v}}^{T}\right]$$

Let $E\left[(B_{k-1}W_{k-1}-O_{k-1}V_{k}^{v}){V_{k}^{v}}^{T}\right]=$0, then the process noise and the observation noise are independent of each other, that is

(53) $$O_{k-1}E\left[V_{k}^{v}{V_{k}^{v}}^{T}\right]=E\left[B_{k-1}W_{k-1}{V_{k}^{v}}^{T}\right]$$

and

(54) $$O_{k-1}=E\left[B_{k-1}W_{k-1}{V_{k}^{v}}^{T}\right]{\left\{E\left[V_{k}^{v}{V_{k}^{v}}^{T}\right]\right\}}^{-1}$$

The new state equation and observation equation of the decoupled system are

(55) $$X_{k}=X_{k-1}\left(F_{k-1}-O_{k-1}H_{k}^{h}\right)+O_{k-1}Z_{k}^{z}+W_{k-1}^{w}$$

(56) $$Z_{k}^{z}=H_{k}^{h}X_{k-1}+V_{k}^{v\;\;}$$

where $W_{k-1}^{w}=B_{k-1}W_{k-1}-O_{k-1}V_{k}^{v}$.

The one-step prediction state vector of the new model is

(57) $${\hat{X}}_{\left.k\right|k-1}^{x}=F_{\left.k\right|k-1}^{x}{\hat{X}}_{k-1}+O_{k-1}V_{k}^{v}$$

The one-step prediction covariance matrix of the state is calculated as follows:

(58) $$\begin{array}{cc} P_{\left.k\right|k-1}^{p}\; & =E\left[\left(X_{k}-{\hat{X}}_{\left.k\right|k-1}^{x}\right){\left(X_{k}-{\hat{X}}_{\left.k\right|k-1}^{x}\right)}^{T}\right]\\ & =E\left[\left(X_{k}-{\hat{X}}_{\left.k\right|k-1}^{x}\right)\left(X_{k}^{T}-{\left({\hat{X}}_{\left.k\right|k-1}^{x}\right)}^{T}\right)\right]\\ & =E\left[X_{k}X_{k}^{T}+{\hat{X}}_{\left.k\right|k-1}^{x}{\left({\hat{X}}_{\left.k\right|k-1}^{x}\right)}^{T}-X_{k}{\left({\hat{X}}_{\left.k\right|k-1}^{x}\right)}^{T}-{\hat{X}}_{\left.k\right|k-1}^{x}X_{k}^{T}\right]\\ & =\left(F_{\left.k\right|k-1}-O_{k-1}H_{k}^{h}\right)P_{k-1}{\left(F_{\left.k\right|k-1}-O_{k-1}H_{k}^{h}\right)}^{T}+Q_{k-1}+O_{k-1}R_{k}^{r}O_{k-1}^{T}-N_{k}O_{k-1}^{T}-O_{k-1}N_{k}^{T} \end{array}$$

where

$$N_{k}=E\left[B_{k-1}W_{k-1}{V_{k}^{v}}^{T}\right]$$

$$R_{k}^{r}=E\left[V_{k}^{v}{V_{k}^{v}}^{T}\right]$$

$P_{\left.k\right|k-1}^{p}$ can be abbreviated as

(59) $$P_{\left.k\right|k-1}^{p}=FP_{k-1}F^{T}+Q_{k-1}^{q}$$

where

$$F=F_{\left.k\right|k-1}-O_{k-1}H_{k}^{h}$$

$${Q_{k-1}^{q}=Q_{k-1}+O}_{k-1}R_{k}^{r}O_{k-1}^{T}-N_{k}O_{k-1}^{T}-O_{k-1}N_{k}^{T}$$

Therefore, the filtering process can be updated as follows:

(60) $$K_{k}=P_{\left.k\right|k-1}^{p}{\left[H_{k}^{h}\right]}^{T}{\left(H_{k}^{h}P_{\left.k\right|k-1}^{p}{\left[H_{k}^{h}\right]}^{T}+R_{k}^{r}\right)}^{-1}$$

(61) $${\hat{X}}_{\left.k\right|k-1}^{x}=F_{\left.k\right|k-1}^{x}{\hat{X}}_{k-1}+O_{k-1}Z_{k}^{z}$$

(62) $$P_{\left.k\right|k-1}^{p}=FP_{k-1}F^{T}+Q_{k-1}^{q}$$

The mean and covariance matrix of the posterior state estimation at time *k* of the system are

(63) $${\hat{X}}_{k}={\hat{X}}_{\left.k\right|k-1}^{x}+K_{k}\left(Z_{k}^{z}-{\hat{Z}}_{\left.k\right|k-1}\right)$$

(64) $$P_{k}=P_{\left.k\right|k-1}^{p}-K_{k}S_{k}K_{k}^{T}$$

#### 4.2.3. A Nonlinear Filtering Process Based on a New Measurement Model of the System

In summary, the UKF filtering algorithm process after denoising processing and removing noise correlation are as follows:

(1) The initialization state ${\hat{X}}_{0}$ and its covariance $P_{0}$, $Q_{0}$, and $R_{0}$.

(2) According to the estimated mean ${\hat{X}}_{k-1}$ and covariance matrix $P_{k-1}$ at time *k*−1, the sigma points and weights are selected. After the nonlinear transformation of the sigma points, the state prediction means ${\hat{X}}_{\left.k\right|k-1}$ and the prediction covariance $P_{\left.k\right|k-1}$ are obtained.

(3) According to the new measurement model of the system, the nonlinear transformation of the sigma point is carried out to obtain the measurement prediction mean ${\hat{Z}}_{\left.k\right|k-1}$, the innovation covariance $S_{k}$, the new step prediction state vector ${\hat{X}}_{\left.k\right|k-1}^{x}$, the prediction covariance matrix $P_{\left.k\right|k-1}^{p}$, and the filtering gain $K_{k}$.

(4) Finally, the mean of state estimation ${\hat{X}}_{k}$ and the covariance matrix $P_{k}$ at time *k* are updated.

Considering that the initial stage of filtering is sensitive to noise, it is easy to cause large errors in the filtering results and this can even lead to filter divergence. Therefore, the above initial positioning value is used as the initial value of UKF for filtering positioning.

### 4.3. Self-Optimizing Parameter Filtering Algorithm Based on Prediction Residuals

The predicted residual statistic is introduced to describe the random influence of the system state. By setting the threshold parameters, the calculated value and estimated value of the covariance matrix are used to judge the prediction error, and the self-optimization parameters are set to optimize the filter gain matrix in real time to enhance the robustness of the system. Set the prediction residual statistics to be

(65) $${∆Z=Z}_{k}^{z}-H_{k}^{h}X_{k-1}$$

Then, the covariance matrix is as follows:

(66) $$P_{k}^{\left(p\right)}=H_{k}^{h}P_{\left.k\right|k-1}^{p}{\left[H_{k}^{h}\right]}^{T}+R_{k}^{r}$$

The self-optimization parameter is set to $\mu_{k}$ and $Tr\left(\cdot \right)$ represents the trace operation. $\mu_{k}$ can be determined according to the following method: when ${\left[∆Z\right]}^{T}∆Z<Tr\left[P_{k}^{\left(p\right)}\right]$, it means that the state prediction error is small, take $\mu_{k}=1$; otherwise, the state prediction error is large, take $\mu_{k}=Tr\left[P_{k}^{\left(p\right)}\right]/\left[{\left[∆Z\right]}^{T}∆Z\right]$. The observation accuracy can ensure that $Tr\left[P_{k}^{\left(p\right)}\right]$ is not too small, so the weight of the observation value with higher accuracy will not be reduced and the filtering error will be reduced.

We use the filter gain matrix to update the parameters and the self-optimization method to filter the noise modeling error. The filter gain is shown as follows:

(67) $$K_{k}=\frac{1}{\mu_{k}}P_{\left.k\right|k-1}^{p}{\left[H_{k}^{h}\right]}^{T}{\left[H_{k}^{h}\frac{1}{\mu_{k}}P_{\left.k\right|k-1}^{p}{\left[H_{k}^{h}\right]}^{T}+{\left(R_{k}^{r}\right)}^{-1}\right]}^{-1}$$

where $\mu_{k}$ is the self-optimization parameter and $0<\mu_{k}\leq 1$.

**Figure 1:**
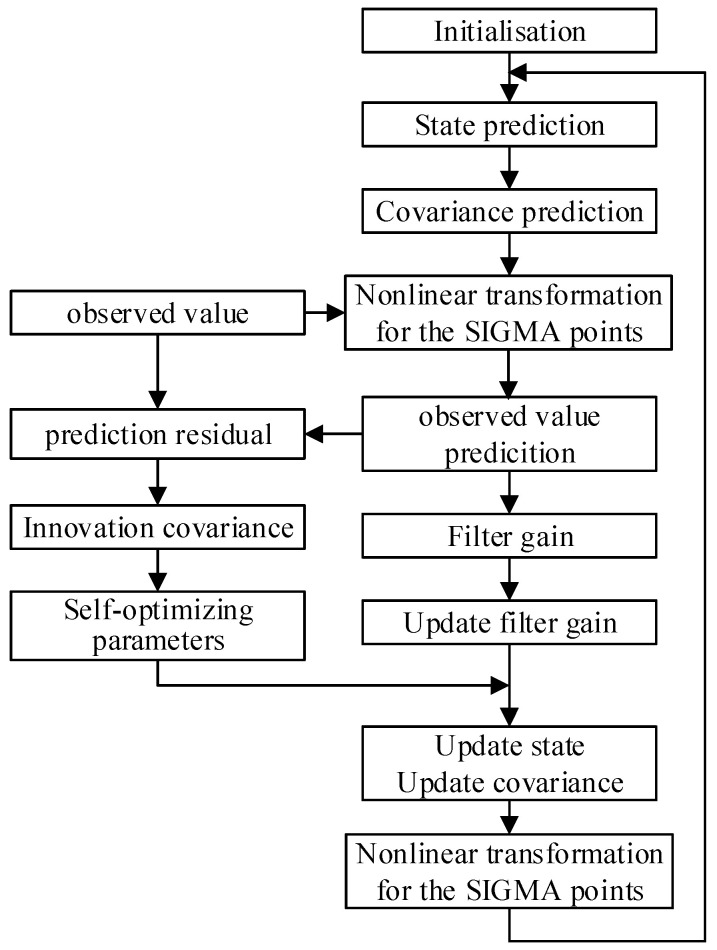
The Kalman adaptive filtering process diagram.

The diagram of the Kalman filter process based on the self-optimization parameters is shown in Figure 1.

## 5. Positioning Performance Evaluation

To evaluate and compare the performance of the positioning algorithm, the relevant evaluation indicators and comparison algorithm are briefly described as follows.

(1) RMSE

The commonly used evaluation indicator representing the estimated performance is the root mean square error (RMSE). If the estimated multipath delay and location are ${\hat{t}}_{y}$ and ${\hat{z}}_{i}$, respectively, the RMSE representing the multipath delay and location estimation performance of the target node are shown in Equations (68) and (69), respectively.

(68) $${RMSE}_{{\hat{t}}_{y}}=\sqrt{\frac{1}{M}\sum_{i=1}^{M}E\left({\left\|{\hat{t}}_{y}-t_{y}\right\|}^{2}\right)}$$

(69) $${RMSE}_{{\hat{z}}_{i}}=\sqrt{\frac{1}{M}\sum_{i=1}^{M}E\left({\left\|{\hat{z}}_{i}-z_{i}\right\|}^{2}\right)}$$

(2) CRLB

The CRLB represents the bound of the estimated error covariance matrix of any unbiased estimate of an unknown parameter vector, which is equal to the inverse of the Fisher information matrix (FIM), and the FIM is created by the probability density function (PDF). The CRLB is briefly described below to compare algorithms.

The actual position coordinate of the target to be estimated is set to $z_{i}={\left[z_{xi},z_{yi},z_{zi}\right]}^{T}$, and the estimated position is marked as $p_{i}={\left[p_{xi},p_{yi},p_{zi}\right]}^{T}$. For non-random vector parameters, the CRLB is expressed as follows:

(70) $$E\left[\left(\left\|p_{i}-z_{i}\right\|\right){\left(\left\|p_{i}-z_{i}\right\|\right)}^{T}\right]\geq {FIM}^{-1}$$

where the PDF of the $t_{ik}$ is

(71) $$p\left(t_{ik}|t_{y},u_{i}\right)=\frac{1}{\sqrt{{\left(2\pi \sigma_{n}^{2}\right)}^{M}}}\exp\left(-\sum_{k=1}^{M}\frac{1}{2\sigma_{n}^{2}}{\left(ct_{ik}-ct_{0}-ct_{y}-\left\|p_{i}-z_{k}\right\|\right)}^{2}\right)$$

The corresponding Fisher information matrix is expressed as follows:

(72) $$FIM=-E\left\{\left[\begin{array}{ccc} \frac{\partial^{2}\ln p}{\partial \tau_{p}^{2}} & \frac{\partial^{2}\ln p}{\partial \tau_{p}\partial p_{xi}} & \begin{array}{cc} \frac{\partial^{2}\ln p}{\partial \tau_{p}\partial p_{yi}} & \frac{\partial^{2}\ln p}{\partial \tau_{p}\partial p_{zi}} \end{array}\\ \frac{\partial^{2}\ln p}{\partial p_{xi}\partial \tau_{p}} & \frac{\partial^{2}\ln p}{\partial p_{xi}^{2}} & \begin{array}{cc} \frac{\partial^{2}\ln p}{\partial p_{xi}\partial p_{yi}} & \frac{\partial^{2}\ln p}{\partial p_{xi}\partial p_{zi}} \end{array}\\ \begin{array}{c} \frac{\partial^{2}\ln p}{\partial p_{yi}\partial \tau_{p}}\\ \frac{\partial^{2}\ln p}{\partial p_{zi}\partial \tau_{p}} \end{array} & \begin{array}{c} \frac{\partial^{2}\ln p}{\partial p_{yi}\partial p_{xi}}\\ \frac{\partial^{2}\ln p}{\partial p_{zi}\partial p_{xi}} \end{array} & \begin{array}{cc} \begin{array}{c} \frac{\partial^{2}\ln p}{\partial p_{yi}^{2}}\\ \frac{\partial^{2}\ln p}{\partial p_{zi}\partial p_{yi}} \end{array} & \begin{array}{c} \frac{\partial^{2}\ln p}{\partial p_{yi}\partial p_{zi}}\\ \frac{\partial^{2}\ln p}{\partial p_{zi}^{2}} \end{array} \end{array} \end{array}\right]\right\}$$

Therefore, the CRLB of time delay and position error variance is shown in Equations (73) and (74), respectively.

(73) $${CRLB}_{\tau_{p}}={\left[{FIM}^{-1}\right]}_{1,1}$$

(74) $${CRLB}_{p_{i}}={\left[{FIM}^{-1}\right]}_{2,2}+{\left[{FIM}^{-1}\right]}_{3,3}+{\left[{FIM}^{-1}\right]}_{4,4}$$

where ${FIM}^{-1}$ is the inverse matrix of FIM, ${\left[{FIM}^{-1}\right]}_{i,j}$ is the element of the matrix ${FIM}^{-1}$ in row *i* and column *j*, and for simplicity, we do not list the closed expression for the full CRLB here. The CRLB of multipath delay and localization performance discussed above will be used in the simulation later in this paper to compare the proposed algorithm with other existing algorithms.

## 6. Numerical Simulation and Analysis

### 6.1. Initial Positioning Simulation

This section evaluates the positioning performance of the proposed positioning algorithm through simulation. Assuming that the measurement error is an independent and identically distributed zero-mean Gaussian vector, the proposed algorithm is run on the target node on the wireless network. At the same time, it is assumed that the time of running the algorithm independently is less than the network data update time; that is, the data from the neighbor nodes are received, and the time is kept unchanged to obtain more accurate and effective positioning results.

The purpose of the simulation setup is to test the relationship between the convergence estimation performance of the proposed algorithm and the measurement error value. The simulation scenario is set as follows: the wireless network consists of nine nodes, of which one target node needs to be located, and the remaining eight anchor nodes are in the area that can normally participate in the positioning. Table 1 shows the true location of nine nodes in the wireless network, where node 6 is the target node to be located, and the remaining nodes are anchor nodes, which can achieve TOA distance measurement with the target node.

The following WLS algorithm and RLS algorithm are described for performance comparison with the algorithm in this paper.

(1) WLS algorithm

It is assumed that there are *N* reference nodes in the network marked as $R_{i}(q_{xk},q_{yk},q_{zk})$, the measurement distance from the reference node to the unknown node $U(z_{xi},z_{yi},z_{zi})$ is $d_{i}$, and the vector to be estimated is from the reference node is denoted as $\theta ={\left[p_{xi},p_{yi},p_{zi}\right]}^{T}$. The measurement equation is

$${\hat{d}}_{i}=d_{i}+v_{i},\;i=1,2,\cdots ,N$$

where $d_{i}=\left\|p_{i}-z_{k}\right\|$, and $v_{i}$ is the measurement error. Assuming that ***h*** is the observation and ***b*** is the observation matrix, the positioning model can be abbreviated as

(75) h=bθ+v

Therefore, the solution of WLS is

(76) $$\hat{\theta }={\left(b^{T}Wb\right)}^{-1}b^{T}Wh$$

where $W={cov}^{-1}\left(v\right)$ is a weighted matrix and is required to be symmetric and positive definite. In practical applications, if the statistical characteristics of errors are unknown, *W* can be set as a unit matrix, and its value can be taken as $W=diag\left\{\begin{array}{ccc} 1/d_{1},1/d_{2} & \cdots , & 1/d_{N} \end{array}\right\}$.

(2) RLS algorithm

Let ${\hat{\theta }}_{k}$ be the estimated value obtained by the least squares algorithm using the first k observation data. The measurement process is modeled as follows:

(77) $$h_{k}=b_{k}\theta_{k}+v_{k}$$

Using the RLS estimation algorithm with a forgetting factor, the measurement error cost function is as follows:

(78) $$J\left(\theta_{k}\right)=\frac{1}{2}\sum_{k=1}^{n}{\lambda^{n-k}\left(h_{k}-b_{k}^{T}{\hat{\theta }}_{k}\right)}^{2}$$

The value of $\theta_{k}$ that minimizes the cost function is the estimated value of ${\hat{\theta }}_{k}$, so the recursive equation of the RLS algorithm with the forgetting factor is as follows:

(79) $${\hat{\theta }}_{k}={\hat{\theta }}_{k-1}+a_{k}\left[h_{k}-b_{k}^{T}{\hat{\theta }}_{k-1}\right]$$

where $a_{k}$ is the recursive gain matrix, $a_{k}=\varphi_{k-1}b_{k}{\left[\lambda I+b_{k}^{T}\varphi_{k-1}b_{k}\right]}^{-1}$, $\varphi_{k}$ is the covariance matrix $\varphi_{k}=\left[I-a_{k}b_{k}^{T}\right]\varphi_{k-1}/\lambda$, and λ is the forgetting factor, $\lambda \in \left(0,1\right)$.

**Figure 2:**
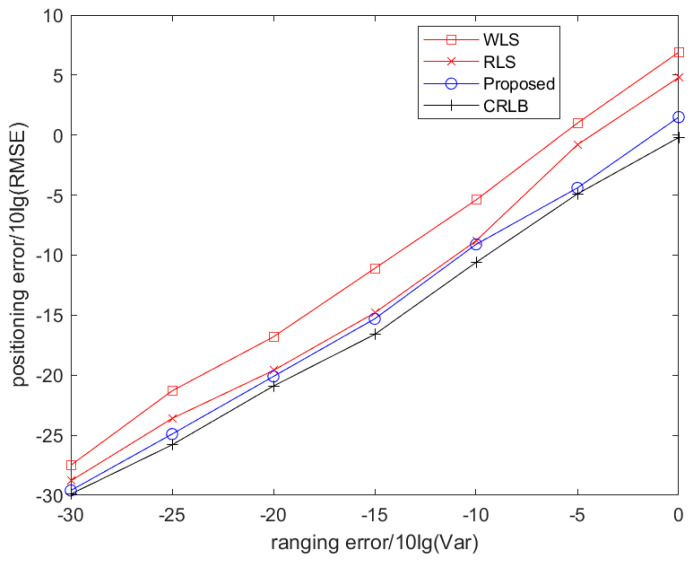
The estimation performance of the algorithm.

When the network is deployed according to Table 1, the location estimation performance of the three algorithms running on the target node 6 is as shown in Figure 2. The curves in the figure are the result obtained by taking the average value of the simulation after 500 times of independent operation.

It can be seen from Figure 2 that all algorithms can locate normally within a certain range of RMSE measurement errors based on TOA. The localization performance of the proposed algorithm is very close to that of CRLB, and better than that of the WLS algorithm and the RLS algorithm.

### 6.2. Filtering Simulation

#### 6.2.1. Experimental Environment

**Figure 3:**
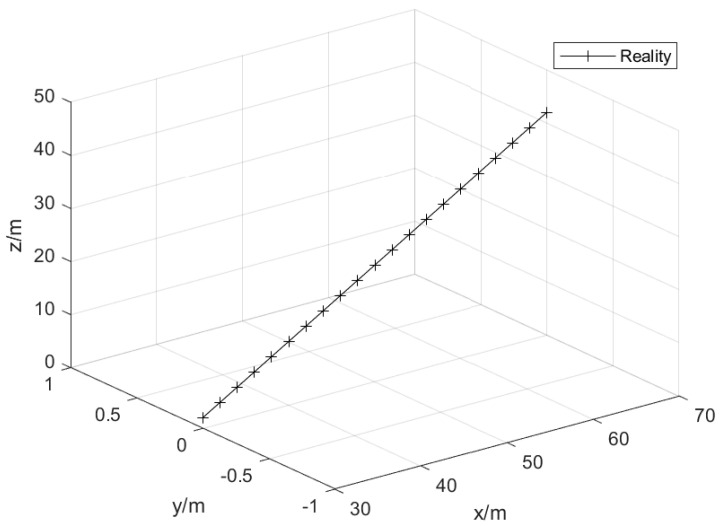
Target motion trajectory.

The target is set to perform uniform linear motion in the three-dimensional plane. The starting position is set to (30, 0, 2), and the unit is m. That is, the real trajectory of the target motion is set to perform uniform linear motion. The motion speed $\left(v_{x},v_{y},v_{z}\right)$ of target is set to (15, 0, 10) and the unit is m/s. The disturbance acceleration variance $\left(a_{x},a_{y},a_{z}\right)$ of the target is $\left(\sqrt{2}/3,\sqrt{2}/3,\sqrt{2}/3\right)$, the unit is ${\left(m/s^{2}\right)}^{2}$, and the sampling period is 0.1 s. The target trajectory is shown in Figure 3.

**Figure 4:**
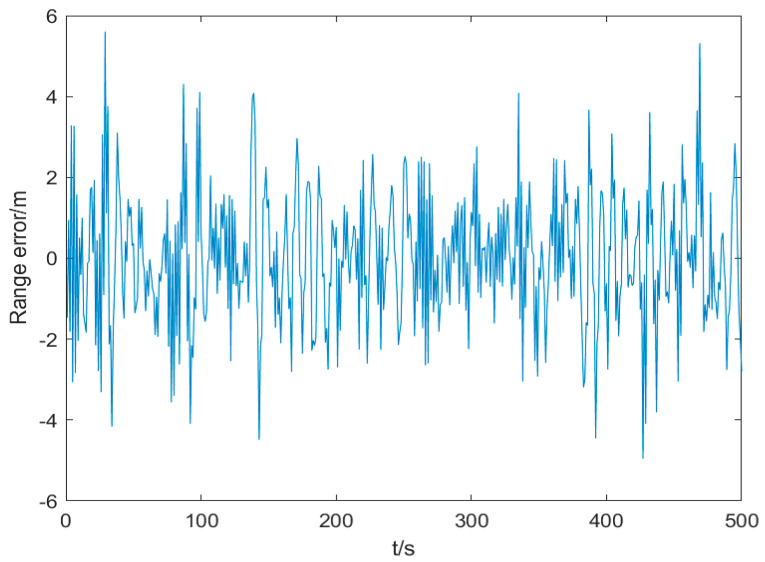
Colored noises caused by TOA measurement.

The TOA ranging error is set as the output of the Gaussian distribution white noise through the first-order linear filter, which can be regarded as the colored noise driven by the white noise. The mean value of the white noise is set to 0, and the standard deviation is 2 m/s. The sampling period is set to 0.1 s, and the total simulation time is 500 s. The standard deviation of TOA colored noise is 1 m, as shown in Figure 4.

#### 6.2.2. Target Observation and Filtering Results

**Figure 5:**
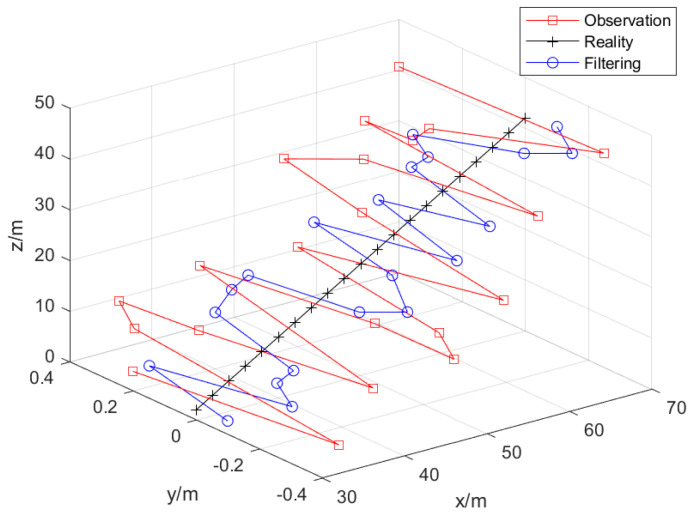
Trajectory of target observation and filtering estimation.

The target is set to perform uniform linear motion in the three-dimensional plane, and the starting position is set to (30, 0, 2), (unit: m). The UKF algorithm based on colored noise processing is used to estimate the trajectory of the target motion. The target and the estimated trajectory are shown in Figure 5, which clearly shows that the estimation of the target motion trajectory by the filtering algorithm in this paper is close to the actual trajectory.

#### 6.2.3. Comparison of Filtering Results Based on White Noise and Colored Noise

**Figure 6:**
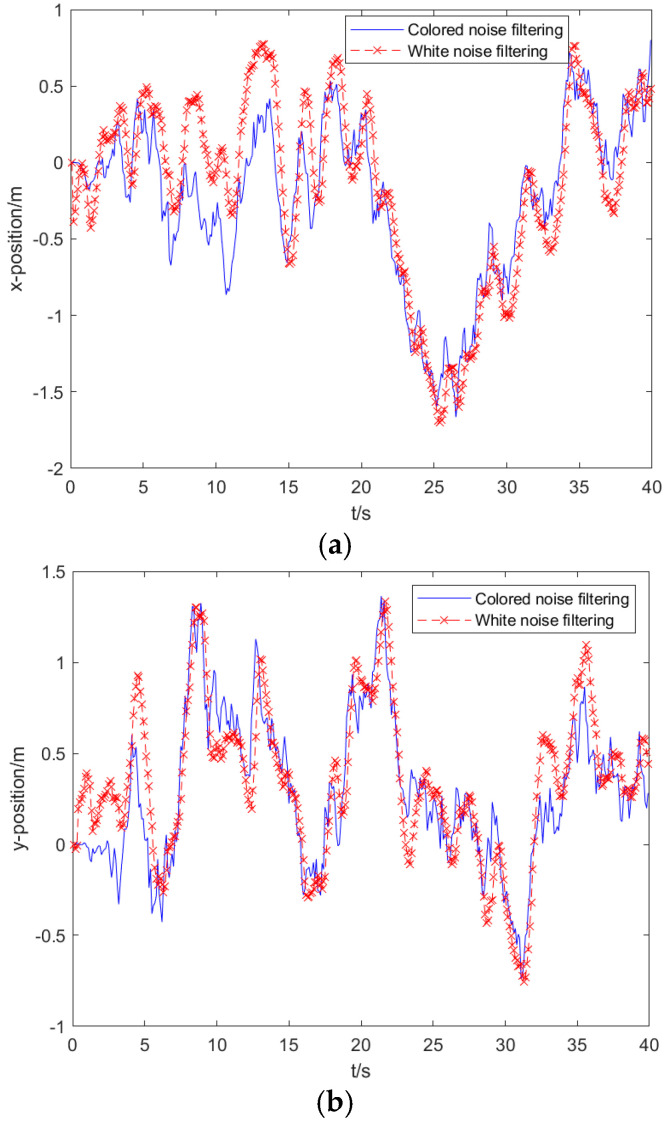

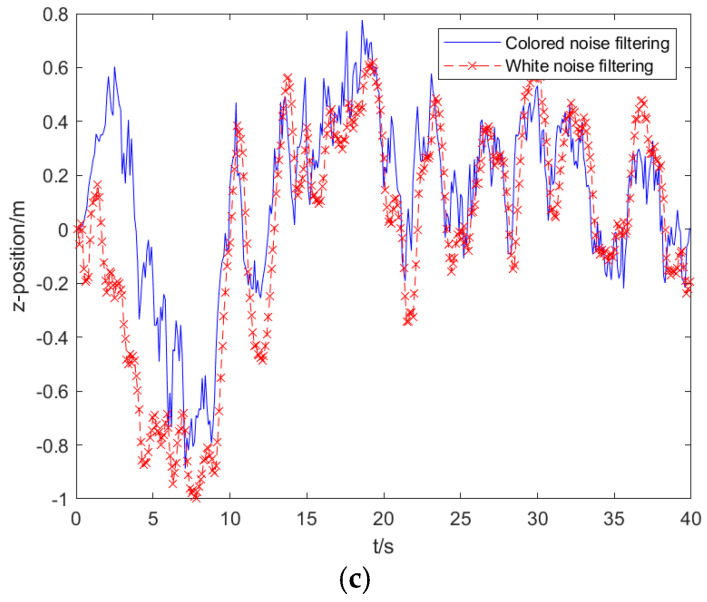
Position error of colored noise and white noise filtering. (**a**) Position error in the *x*-axis direction. (**b**) Position error in the y-axis direction. (**c**) Position error in the z-axis direction.

The sampling period is set to 0.1 s, and the filtering estimation results of white noise and colored noise are compared. The estimation results of the two algorithms are shown in Figure 6 by the position errors in the x-axis direction, the y-axis direction, and the z-axis direction, respectively. The phase performance of filtering errors is more complex, but the overall trend shows that the filtering estimation accuracy after colored noise processing is higher. The mean and standard deviation of the errors of the two algorithms in the three directions of the rectangular coordinate axis are shown in Table 2, which shows that the filtering performance of the processed colored noise is high, and the standard deviation has been maintained at a low level.

#### 6.2.4. Prediction Error and Filtering Error

**Figure 7:**
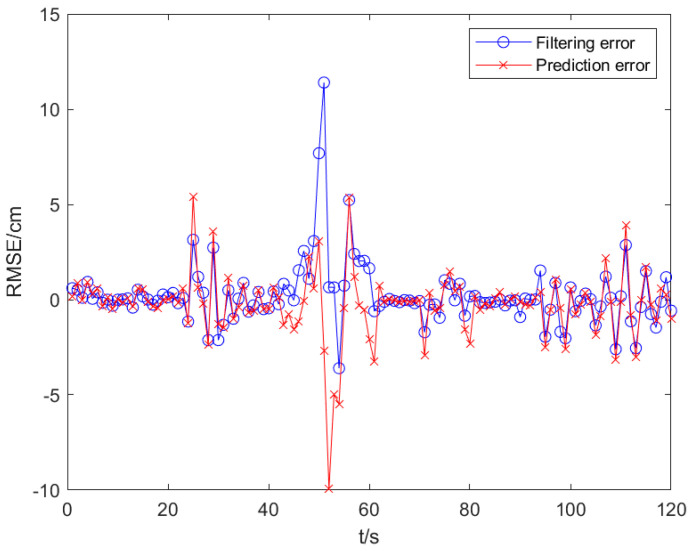
Position error of prediction and filtering.

Set the target to perform uniform linear motion. The starting position of the target is now set to (30, 0, 2), (unit: m). The simulation results show that the standard deviations of the prediction error and the filtered position error are 0.817 and 0.362, respectively, under the condition of colored noise. The position error curves in both cases are shown in Figure 7.

#### 6.2.5. RMSE Error and Analysis Based on Colored Noise Processing

**Figure 8:**
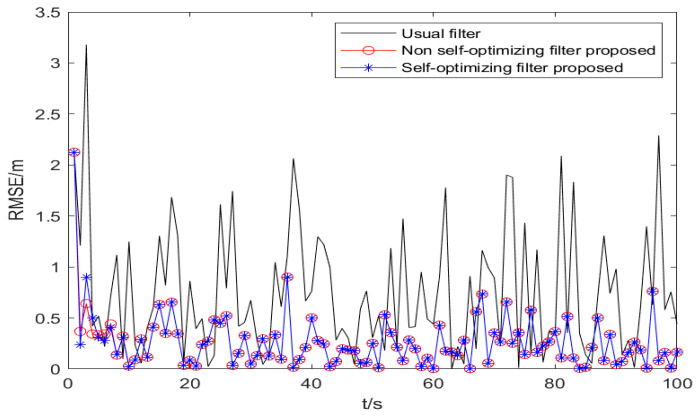
RMSE positioning error of three filtering algorithms.

The RMSE is used to calculate the error between the target estimation and the actual position. Under the premise that the observation noise is a colored noise vector, the environmental parameters are set as the same as the previous uniform linear motion, and the basic UKF, the non-self-optimizing UKF, and the self-optimizing UKF algorithm are run, respectively. The results are shown in Figure 8.

**Figure 9:**
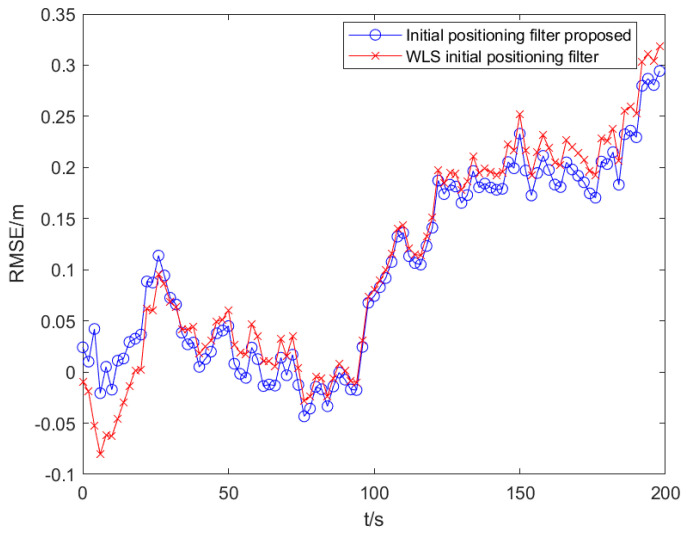
Filter positioning error of different initial positioning.

In the total simulation time of 100 s, the mean values of RMSE of the target are 0.32 m, 0.35 m, and 0.67 m, respectively. The results of Figure 9 show that in the positioning based on TOA ranging, the observation with colored noise will seriously affect the positioning accuracy, and the basic KF algorithm has large filtering errors and a poor stability. The filtering algorithm in this paper can reduce the positioning error caused by colored noise and has a good stability. The filtering algorithm in this paper improves the filtering accuracy by 52.23% compared with the basic KF algorithm.

#### 6.2.6. Filtering Results and Analysis Under Different Initial Positioning Conditions

In the actual ranging-based positioning system, there is always an error between the given initial state value and the optimal initial value required for filtering, which will affect the overall filtering performance. Choosing an accurate initial value of the filter can not only eliminate the adverse effects of the initial value error on the filtering process but also ensure the fast convergence of the filtering results.

The experimental environment is set as follows: the target moves in a uniform linear motion in the three-dimensional plane, and the starting position of the target is accurately estimated by the TOA measurement of the M anchor nodes received by Algorithm 1 as $\left({\hat{x}}_{0},{\hat{y}}_{0},{\hat{z}}_{0}\right)$, and other settings remain unchanged.

The definition of RMSE is used to calculate the error between the target estimation and the actual position on the x-axis and the y-axis, respectively. Under the premise that the observation noise is a colored noise vector and the parameter setting environment is the same, the self-optimized UKF algorithm based on WLS initial positioning and the self-optimized UKF algorithm based on the parameter analysis initial positioning in this paper are run, respectively, as shown in Figure 9.

It should be noted that the error of the initial positioning in the initial stage is smaller than that of other algorithms without initial positioning, and the error in the steady state is slightly smaller than that of other algorithms without initial positioning.

From the simulation results of Figure 9, in the total simulation time of 200 s, the mean values of RMSE obtained by the two algorithms are 0.051 m and 0.072 m, respectively, and the filtering accuracy is improved by 28.57%. The standard deviation of filtering is 0.418 m and 0.573 m, respectively, which shows that the algorithm in this paper not only has a high positioning accuracy, but also shows that the filtering performance of the initial positioning in this paper is high, and the standard deviation has been maintained at a low level.

The above statistical data can demonstrate that under the same conditions, the algorithm proposed in this paper selects more accurate initial filtering values that are closer to the optimal initial values required for filtering, which can simulate the negative impact of initial value errors on filtering performance. However, based on WLS positioning, there is a significant error between the initial values and the optimal filtering initial values. Therefore, the algorithm proposed in this paper can achieve a higher filtering accuracy, as evidenced by the results shown in Figure 9.

#### 6.2.7. Computation Time

To analyze and compare the complexity of the algorithms proposed in this article, we calculated the average running time of the algorithms. The data simulation processing platform consists of a PC with the following parameters: Intel (R) Core (TM) i7-8700 CPU @ 3.20 GHz, 16.0 GB RAM, 64-bit operating system based on x64 processor, and MATLAB 2019A as the software testing environment for executing the simulation process. The simulation scenario is described in Section 6.2.6. The self-optimized UKF algorithm based on WLS initial positioning and the self-optimized UKF algorithm based on parameter analysis initial positioning in this paper are independently run 50 times each. The average positioning running time is shown in Table 3. The positioning running time of the two algorithms in the self-optimizing UKF algorithm stage is the same. In the initial positioning stage, the initial positioning algorithm proposed in this paper improves the positioning accuracy, but at the same time, it also comes at a slightly higher cost of increased running time. Therefore, under the condition of limited and enough computing power, it is worthwhile to use the algorithm proposed in this paper for higher accuracy positioning.

## 7. Conclusions

In this paper, a gain-adaptive two-stage fusion filtering localization algorithm based on prediction residuals is proposed. Firstly, based on the Gaussian noise observation model, the pseudo-linearization equations from TOA measurements and multipath time delays are derived using the nonlinear system parameterization method. The WLS criterion and effective analytical calculation are used to obtain an approximate analytical solution that approximates the CRLB of the target position estimation, which is used as a Kalman filter after the initial positioning input. In the final positioning stage, the colored noise measurement information is processed by reconstructing the Gaussian distribution white noise statistics, and the new measurement model is derived. Subsequently, the Kalman filtering algorithm is used to implement multi-source information fusion, and the new observation is received for filtering error correction. This study explores the construction of measurement models based on time of arrival (TOA) measurement and multipath delay to achieve localization. In order to compensate for modeling errors caused by noise, observation values are introduced to predict residual statistics, covariance matrix tracking operations, and gain matrices in self-optimizing filters to obtain the accurate position of the target, thereby enhancing the filtering performance of the localization system for time measurement errors. The positioning effect of the proposed algorithm is verified by simulation experiments. The experimental results show that compared with other initial positioning algorithms, the average positioning accuracy of the fusion algorithm in this paper is improved by 28.57%, exhibiting better accuracy and filtering stability. In this work, additive colored noise in the localization model is analyzed and whitened, enabling the system to meet the Kalman filter requirements and achieve high-precision estimation. However, non-additive (e.g., multiplicative) effects arise in practical positioning scenarios. Future work will focus on analyzing and whitening non-additive noise to further improve the Kalman filtering performance. Furthermore, extending the proposed framework to handle complex motion trajectories (e.g., circular or nonlinear patterns) represents a critical direction for real-world applications, as such trajectories inherently exist in cluttered environments. This extension will require the development of adaptive motion pattern models and corresponding uncertainty quantification methods to address dynamic constraints under non-ideal path conditions.

## Figures and Tables

**Table 1:** Units for magnetic properties.

Coordinates	1	2	3	4	5	6	7	8	9
x	−300	0	300	−300	0	300	−300	0	300
y	300	300	300	0	0	0	−300	−300	−300
z	−300	−300	300	300	0	0	300	0	−300

**Table 2:** The error mean and standard deviation of the two algorithms in three directions.

Performance of Error (m)	Gaussian White Noise			Processed Colored Noise		
	X-Axis	Y-Axis	Z-Axis	X-Axis	Y-Axis	Z-Axis
Mean	−0.3857	0.2352	−0.6384	−0.2367	−0.0153	−0.4231
Standard deviation	0.6652	0.6256	0.6132	0.4428	0.4432	0.4284

**Table 3:** Average running time.

Algorithm	WLS Initial Positioning Filter	Initial Positioning Filter Proposed
Time (ms)	10.78	11.85

## Data Availability

The data supporting the findings of this paper are available from the corresponding author upon reasonable request.
